# Electrophysiology in Functional Movement Disorders: An Update

**DOI:** 10.5334/tohm.793

**Published:** 2023-12-26

**Authors:** Nitish Kamble, Pramod Kumar Pal

**Affiliations:** 1 Departments of Neurology, National Institute of Mental Health & Neuro Sciences (NIMHANS), Hosur Road, Bengaluru-560029, Karnataka, India

**Keywords:** Bereitschaftspotential, Electromyography, Electrophysiology, Functional dystonia, Functional myoclonus, Functional tremors, Transcranial magnetic stimulation

## Abstract

**Background:**

Functional movement disorders (FMD) are a diagnostic and therapeutic challenge, both to the neurologist and psychiatrists. The phenomenology is varied and can present as tremors, dystonia, jerks/myoclonus, gait disorder, other abnormal movements or a combination. There has been an increase in the use of electrophysiological studies that are an important tool in the evaluation of FMDs.

**Methods:**

We searched the database platforms of MEDLINE, Google scholar, Web of Sciences, Scopus using the Medical Subject Heading terms (MeSH) for all the articles from 1st January 1970 till November 2022. A total of 658 articles were obtained by the search mechanism. A total of 79 relevant articles were reviewed thoroughly, of which 26 articles that had electrophysiological data were included in the present review.

**Results:**

Variability, distractibility and entertainability can be demonstrated in functional tremors by using multichannel surface electromyography. Voluntary ballistic movements tend to decrease the tremor, while loading the tremulous limb with weight causes the tremor amplitude to increase in functional tremor. Presence of Bereitschaftspotential demonstrates the functional nature of palatal tremor and myoclonus. Co-contraction testing may be helpful in differentiating functional from organic dystonia. The R2 blink reflex recovery cycle has been found to be abnormally enhanced in organic blepharospasm, whereas it is normal in presumed functional blepharospasm. Plasticity is found to be abnormally high in organic dystonia and normal in functional dystonia, in addition to enhanced facilitation in patients with organic dystonia.

**Conclusions:**

Electrophysiological tests supplement clinical examination and helps in differentiating FMD from organic movement disorders.

## Introduction

Functional movement disorders (FMDs) are one among the commonly encountered disorders in neurological practice that forms a part of the spectrum of functional neurological disorders [1]. Earlier the diagnostic criteria required the presence of a clear psychological or emotional stress in establishing the diagnosis. Now there is an emphasis for positive clinical diagnostic criteria supplemented by electrophysiology [2]. FMD’s are a diagnostic and therapeutic challenge, both to the neurologist and psychiatrists [3, 4]. The underlying cause of the disorder is poorly understood and it is possible that psychological trauma, genetic susceptibility, environmental and other factors may play a role. The phenomenology is varied and can present as tremors, dystonia, jerks, gait disorder, other abnormal movements or a combination. The neurologic symptoms are incongruent with known neurologic disease but are a cause of distress and/or psychosocial impairment [5]. The diagnosis of FMD is essentially clinical [6]. The clinical pointers towards a functional etiology include abrupt or sudden onset of movement disorder, presence of antecedent illness, changing phenomenology, bizarre movements, paroxysmal symptoms, presence of multiple phenomenologies, deliberate slowness of movements, presence of variability, entrainment, suggestibility and distractibility of the movements [7, 8].

There has been an increase in the use of electrophysiological studies that are an important tool in the evaluation of FMDs. These tests provide valuable information that help in differentiating it from organic movement disorders. Electrophysiological evaluation has been well established for functional tremors, myoclonus and least for dystonia. The choice of electrophysiological tests depends on the nature or phenomenology of FMD.

Functional tremor (FT) is the most common form and represents about 50% of all FMDs [9]. FMDs are a great concern to the patient as it reduces the quality of life. The various electrophysiological tests provide an objective criterion of FMD. The electrophysiological battery includes surface electromyography (sEMG) and accelerometer, pre-movement potentials, electroencephalography (EEG), jerk locked back averaging, somatosensory evoked potentials and transcranial magnetic stimulation (TMS).

This review provides an update on the role of electrophysiological methods in FMDs.

## Methods of literature search

**Figure 1:**
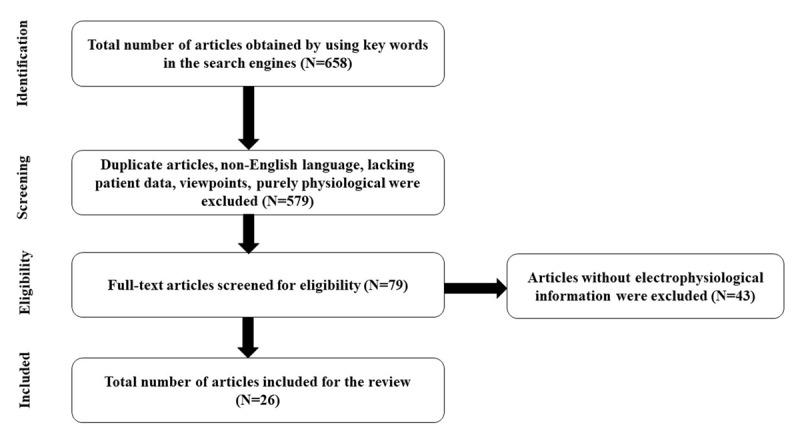
Literature search methods.

We searched the database platforms of MEDLINE, Google scholar, Web of Sciences, Scopus using the Medical Subject Heading terms (MeSH) “Psychogenic movement disorders”, “Functional movement disorders”, And “electrophysiology of functional movement disorders,” “neurophysiology of functional movement disorders,” electrophysiology of psychogenic movement disorders,” “neurophysiology of psychogenic movement disorders,” “functional/psychogenic tremors/dystonia/tics/parkinsonism/myoclonus” for all the articles from 1st January 1970 till November 2022. A total of 658 articles were obtained by the search mechanism. The studies were reviewed critically with respect to title, authors, type, and sample size by thorough screening of the abstracts. Clinical studies of electrophysiology in functional or psychogenic movement disorders were included for the review. Duplicate articles were identified and removed. Those articles whose abstract were lacking were assessed based on the title. Strict inclusion and exclusion criteria were applied for selecting the article. Articles that were excluded were: viewpoints, lacking patient data, non-english language and purely physiological. Based on these a total of 79 relevant articles were reviewed thoroughly, of which there were 33 original articles, 2 case reports and 44 review articles. Finally, 26 articles that had electrophysiological data were included in the present review (Figure 1).

## Results

Various electrophysiological tools have been employed for studying functional tremor, myoclonus and dystonia. These include the following.

### Surface Electromyography (sEMG)

Multichannel sEMG is a non-invasive method of recording the muscle EMG by placing surface electrodes on the skin overlying the muscles likely causing the abnormal movements. It can hence be used to study the EMG signal in patients with tremors, myoclonus and dystonia. The frequency, duration and pattern of EMG bursts helps in characterizing the nature of tremor and the possible diagnosis. Preferably, the recording should include both agonist and antagonist muscles and also distal and proximal muscles in case of complex movements [10]. The active and reference electrodes are placed in a belly tendon montage. In case of larger muscles, the two electrodes can be placed on the muscle belly separated by at least 3 cms. The recommended amplifier settings include a sampling rate of at least 1000 Hz with bandpass filters between 20 – 500 Hz. Variability, distractibility and entrainment can be demonstrated by this method in functional tremors. A proper baseline recording should be done prior to performing any manoeuvres [10]. The amplitude, rhythmicity, frequency and burst duration of the sEMG signals are analysed. sEMG recording is limited by its inability to record the activity of deep muscles and false recording from the adjacent muscles (crosstalk). The details of the technique can be found in a publication by Schwingenschuh P et al. [11]. They advocated the recordings be performed with (a) arms relaxed and hands hanging freely from the arm rest, (b) with arms/wrists outstretched at shoulder level without and (c) with loading a 500 g weight to the wrists during the finger tapping and ballistic movement task. In the tapping task, the patients are instructed to tap using the index finger of the less-affected hand and to time with a metronome at a frequency of 1, 3 and 5 Hz. In the ballistic movement task, the subjects are asked to point with the index finger of an outstretched arm and point as fast as possible towards the abruptly changing position of the examiners index finger. Simultaneous EMG and accelerometery recordings are performed continuously from the more affected limb during the finger tapping and ballistic movement tests [11]. They calculated a sum score for all performed tests (maximum of 10 points) and used a cut-off score of 3 points for a diagnosis of laboratory-supported functional tremor. This battery has yielded a good interrater reliability and test-retest reliability. There was a significant difference between the average score for patients with functional tremor (3.661.4 Points) and patients with organic tremor (1.060.8 points; P < .001), and this yielded a test sensitivity of 89.5% and specificity of 95.9% [11]. The response to tapping was noted for entrainment, tremor suppression or pathological frequency shift. The latter is defined as a frequency shift of at least 19%, 26.9% and 25.7% during contralateral finger tapping at 1, 3 and 5 Hz respectively.

### Accelerometers

The frequency and amplitude are best recorded by accelerometery. Accelerometers are lightweight materials that are applied to the affected parts such as the fingers, the dorsum of hands, head etc. and are connected to the computer interface for accurate characterization of the tremors [12]. These are electromechanical transducers that produce electrical / voltage output at their terminals that is proportional to the acceleration to which it is subjected. They measure the static (force of gravity acting on the body part) and dynamic acceleration forces (movement caused by the tremor). Among the several kinds of accelerometers, the two most frequently used are based on the piezoelectric effect or on the capacitance variation. The first type uses a microscopic crystal which is sensitive to the acceleration forces, generates a measurable voltage. In the second type, the acceleration forces modify the capacitance of these structures in the accelerometer creating a variation that is transformed into a measurable voltage [13]. Accelerometers can be single or triaxial. Triaxial accelerometers record the movements in three mutually perpendicular axes simultaneously. These provide more information compared to the single axis accelerometers. Hence, triaxial accelerometers should be considered for measuring the tremor with its z-axis being placed perpendicular to the most prominent tremor axis. However, if the accelerometer has only one axis (single), then it should be set perpendicular to the axis of the tremor. The data obtained is analysed using the fast Fourier transformed (FFT) technique.

### Tremor analysis

Tremor analysis provides an objective characterization of tremor and complements the clinical examination in patients with tremors [14]. It also helps in differentiating the type of tremor and the number of oscillators generating the tremor. In order to derive this information from the tremor, a triaxial accelerometer is very helpful [15]. The accelerometers are attached on the fingers, dorsum of the hand etc. with the recording axis aligned to the direction of the dominant tremor. The filter settings used for accelerometer recordings include 2 Hz high pass and a 30 Hz lowpass [14, 16].

The frequency of the tremor is the most commonly investigated parameter following EMG or accelerometer recordings. Functional tremors usually have a frequency between 4–9 Hz [17]. The frequency is compared during rest, posture or action. The variability in the frequency can be quantified using various methods that includes frequency spread, tremor consistency (proportion of the time spent at the modal frequency), tremor stability (area under the curve between two vertical lines at half peak power of the frequency spectrum), power spectrum variability (power mean-deviation of the frequency spectrum), Tremor Stability Index (absolute interquartile range of the cycle-by-cycle variation in tremor frequency of the accelerometer axis) [17, 18, 19]. The EMG burst duration correlates moderately with the tremor frequency [20]. There is usually no significant difference in the burst duration between ET, PD and functional tremors [18].

The amplitude of the tremor shows great variation and is dependent on the EMG properties and does not show significant difference between functional and other organic tremors [21].

The EMG and accelerometer data acquired is in the “time domain” that is shown as a change in voltage with time and change in acceleration with time respectively. However, the tremor analysis is possible when the data is in the “frequency domain”. As there are multiple frequencies in the EMG signal, extracting the frequency of the tremor is especially challenging. Hence, the time domain is converted to the frequency domain using the fast Fourier transformation [22]. The raw EMG data is rectified and smoothed before conversion to “frequency domain.” However, it is also important to look at the time domain for irregularity, an abrupt change of tremor frequency and the interaction of the EMG in the agonist-antagonist pairs.

The Fourier transformation analysis takes into account all the frequencies present in the EMG signal and presents it as a series of convolutions. The amplitude of the convolutions is expressed as a “power” which is the amplitude squared. Each of the convolutions with different frequencies is plotted by power plot that provides the relative power of the different frequencies within the recorded signal [14]. If the tremor characteristics are changing over time, then the tremor recording is fragmented into small segments which are analysed separately using Fourier transformation. The results are averaged and expressed as a power spectral plot that shows the change in power of each frequency over time.

The half-power bandwidth is an important measure of tremor rhythmicity and is defined as the width of the spectral peak at one-half the peak amplitude in the power spectrum or at 0.707 peak amplitude in the amplitude spectrum [15]. The half-power bandwidth becomes increasingly narrow as the tremor becomes finely “tuned” to a single frequency [23]. It is narrow in PD tremor and is wider/larger in postural tremors and dystonic tremor.

### Coherence analysis

This is a type of analysis done to look for coherence between the two channels. Coherence analysis expresses the similarity in frequency of two signals. It is usually done to find the correlation between the two EMG signals. It should be done between the EMG signals from both sides to determine whether the tremor is generated by a single or multiple oscillators. It should not be done between an EMG and accelerometer signal as it could suggest false coherence due to transmission of oscillation.

It uses a mathematical tool to examine the relation between the two signals (EEG or EMG signals) in the frequency domain and their dependency on each other. Coherence analysis can be estimated in the sEMG recordings by performing a fast Fourier transformation of the rectified EMG signal. The values range from 0 to 1. A value of 1 reflects a high coherence suggesting a common generator of the tremor [24].

The EMG signals are filtered (50–800 Hz) and analog to digital (A/D) converted (1000 Hz) which is in a computer and full wave rectified for further analysis. The duration of each record is usually 30 sec. The data analysis is done on linear spectral methods and power spectra are estimated for every muscle and the spectra of coherence is calculated for all muscle combinations. Coherence analysis is then used to measure the extent to which signals are correlated in the frequency domain [24].

### Wavelet coherence analysis

Wavelet coherence analysis is a useful additional tool and superior to standard coherence analysis as it helps in discriminating functional from organic tremor with high accuracy [25]. This method detects variations in coherence and differences in phase between the EMG signals and helps to differentiate organic tremor from functional tremor and is more precise than conventional coherence analysis [25].

The parameters that have the most discriminative values are the percentage of time with significant coherence (PTSC) and the number of periods without significant coherence (NOV).

### Bereitschaftspotential (BP)

The simultaneous recording of the EMG and EEG can be analysed by using back averaging technique. The analysed data suggests whether the EMG activity is preceded by EEG activity. These potentials are called movement related cortical potentials (MRCP) and precede the onset of self-initiated voluntary movement. Bereitschaftspotential (BP) is one of the most important electrophysiologic tools used to differentiate functional from organic myoclonus. It is the cortical activity that is picked up prior to the onset of voluntary movement and represents movement preparation. It is seen as a slow rising potential that is maximum at the vertex and begins around 1500–1000 msec prior to the movement [26]. It is measured by back averaging the EEG epochs, a technique that improves the signal-to-noise ratio. The principle of recording BP is the technique of averaging the brain’s EEG activity over multiple trials with the movement acting as the trigger. The EEG preceding the movement artifact is averaged over multiple trials of self-initiated movements and BP is identified as a slow rise in the EEG that is seen prior to the movement onset. BP recorded before the EMG activity in patients with functional myoclonus is similar in appearance to that of normal voluntary movements [9]. It was Kornhuber and Deecke who described BP for the first time in 1964. The presence and duration of BP is potentially useful to differentiate between organic and functional myoclonus [27]. Two components of BP can be recognised, the early (1500 to 1000 msec) and late BP (1000 to 500 msec) [27]. Early BP is linked to motivational, intentional, timing and selection of the movement, whereas the late BP is concerned mainly with motor execution and performance [28].

### R2 Blink Reflex

The blink reflex is elicited by electrical stimulation of the supraorbital nerve and recording the electromyographic response from bilateral orbicularis oculi. An ipsilateral response (R1) and a late bilateral response (R2 and R2’) are recorded. The excitability of the blink reflex neural circuitry is determined by estimating the R2 recovery curve and R2 index. The supraorbital nerve is stimulated with subthreshold intensity initially and gradually the intensity is increased till a small R2 is recorded (R2 threshold). Two consecutive electrical stimuli (conditioning and test stimuli) of equal intensity 2–3 times R2 threshold) are delivered to the supraorbital nerve at different interstimulus intensities (ISIs) of 5000, 2000, 1000, 750, 500, 250 and 100 msec [29]. Six successive responses are recorded and R2 recovery curves are obtained by plotting the size of the test response as a percentage of the conditioning response at each interstimulus interval. The R2 recovery index is calculated as the mean recovery of peak amplitude values at various ISIs and the most sensitive ISIs being 500 and 250 msec [30]. The R2 recovery index is mainly used to differentiate organic versus functional blepharospasm.

### Transcranial magnetic stimulation (TMS)

Transcranial magnetic stimulation is a non-invasive type of brain stimulation that is based on Michael Faraday’s principle of electromagnetic induction. It was first introduced by Anthony Barker and colleagues in 1985. It stimulates the superficial cortical neurons using a powerful transient magnetic field which secondarily induces electric currents in the brain and propagates along the corticospinal volleys [31]. Using different TMS protocols (single and paired pulse stimulations), various parameters such as resting motor threshold (RMT), cortical silent period (CSP), short interval intracortical inhibition (SICI), intracortical facilitation (ICF) etc. can be studied. This provides a valuable information about the cortical excitability, inhibitory and excitatory properties of the brain. CSP and SICI are mediated by GABA-A and GABA-B receptors respectively, whereas ICF is mediated through glutamatergic receptors [32].

Systematic studies of TMS in FMD are lacking. TMS studies have shown reduced intracortical inhibition and silent period in both organic and functional dystonia [33, 34]. However, only small rTMS studies in FMD have demonstrated transient therapeutic potential in patients with FMD [35, 36, 37, 38].

### Electrophysiology in the diagnostic criteria of FMD

The diagnosis of FMD is primarily based on clinical history and demonstration of positive clinical signs. Earlier the diagnosis of FMD was viewed as a “diagnosis of exclusion.” However, the diagnostic criteria have evolved over time with a better understanding of the disease pathophysiology. The initial diagnostic criteria were suggested by Fahn and Williams who categorized FMD in to clinically documented, clinically established, probable and possible FMD [39]. This criterion was mainly developed for functional dystonia which was later applied to other FMDs also. This was later modified by Shill and Geber who categorized FMD in to clinically proven, clinically definite, clinically probable, and clinically possible [40]. This criterion provided a sensitivity of 83% and a specificity of 100% in identifying “clinically probable” cases, while for “clinically possible” or greater, the sensitivity was 97% and specificity was 96% [40]. The Shill-Gerber criterion has a low inter-rater reliability for both the probable and possible categories. Brown and Thompson also used electrophysiological methods in diagnosing functional myoclonus, tremors and dystonia [41]. Gupta and Lange included “laboratory supported definite” diagnostic category based on electrophysiologic testing for the diagnosis of FMD [42]. Schwingenschuh et al. reported the sensitivity and specificity of various electrophysiological tests to distinguish functional from organic tremor. They proposed that a battery of electrophysiological tests that included performance at tapping task at different frequencies, changes with ballistic movements, tonic coactivation, coherence analysis and increase in tremor amplitude with weight loading could identify functional tremor with greater sensitivity (89.5%) and specificity (95.9%) [11].

## Discussion

### Functional Tremors

Functional tremors can involve any body part and can occur at rest or on posture. Clinically the tremors are variable in frequency, amplitude and pattern, and multichannel sEMG is the initial electrophysiological test recommended in these patients. There are numerous positive signs that can be elicited in patients with functional tremor that includes distractibility, variability, entrainment, coactivation sign, response to weight load and pause with ballistic movement. Among them, distractibility is most observed whereas entrainment is infrequently observed. Variability, distractibility and entrainment are the most important signs clinically that are most often sufficient to differentiate functional from organic tremors [43].

During the evaluation of functional tremor, a multi-channel sEMG and at least two accelerometers are frequently used. The EMG and accelerometer data must be acquired for a sufficient length of time, up to few minutes. EMG and accelerometers are placed over the agonist and antagonist muscles causing the tremor after careful clinical evaluation. Tremor is recorded in different positions such as at rest, on posture or activity, weight loading, distraction by mental task and/or voluntary movements of the unaffected limb.

The key diagnostic feature to differentiate between organic and functional tremor is the response to distraction. Distraction can be achieved by a variety of motor and cognitive tasks, which can be demonstrated objectively by the electrophysiological tests (Videos 1 and 2). Tremor frequency of 6 – 11 Hz using the frequency analysis of the EMG recordings suggests more of functional tremor [44, 45, 46]. However, this frequency may also be observed in organic tremors. The EMG and accelerometric data acquired is analysed for the pattern and duration of EMG bursts, tremor amplitude and frequency.

**Video 1:** Demonstration of distractibility while performing motor tasks (voluntary movements of the left hand abruptly stop the tremor in the right hand).

**Video 2:** Demonstration of distractibility while performing mental tasks (counting numbers reduces the frequency and amplitude of the tremor in the right hand).

Patients with essential tremors and enhanced physiological tremors usually demonstrate synchronous EMG bursts, whereas patients with PD demonstrate alternating tremor. Patients with FMD also demonstrate alternating pattern. The EMG burst duration is usually prolonged in functional tremors (>70–80 msec) compared to organic tremors. In addition, varying EMG burst duration is also a typical feature of functional tremor. However, patients with dystonic tremors also can have prolonged and varying EMG burst duration [47]. The tremor amplitude varies in both organic and functional tremors and hence is usually not helpful in differentiating the two conditions. Variation in tremor amplitude can be observed on distraction in functional tremor. Peripheral weight loading can increase the tremor amplitude in functional tremors. The tremor frequency is low and varying in functional tremors. The tremor frequency also changes with distraction, voluntary movements of the unaffected limb, dissipated tremor frequency spectrum, high coherence between the “involuntary” and “voluntary” movements [47]. Among the variability in amplitude and frequency observed in both functional and some organic tremors, variability in distribution appears to differentiate FMD from organic tremors.

Co-activation sign can be demonstrated electrophysiologically in functional tremor, wherein there is tonic co-activation of both the agonist-antagonist muscles at the onset of tremor preceding the reciprocal alternating EMG bursts. In this sign, both the agonist and antagonist muscles contract simultaneously approximately 300 msec before the onset of tremor producing a clonus state leading to the generation of tremor [48]. This sign is not observed in patients with organic tremor.

**Figure 2:**
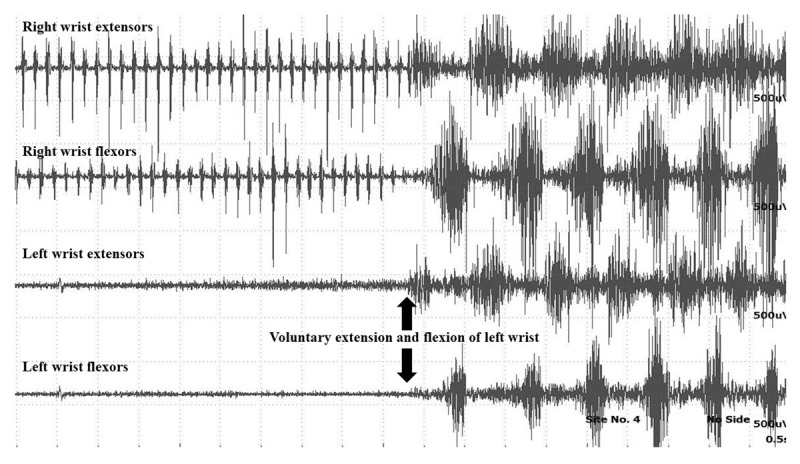
Multichannel surface EMG showing entrainability in a patient with unilateral upper limb functional tremor (from authors’ archive).

**Figure 3:**
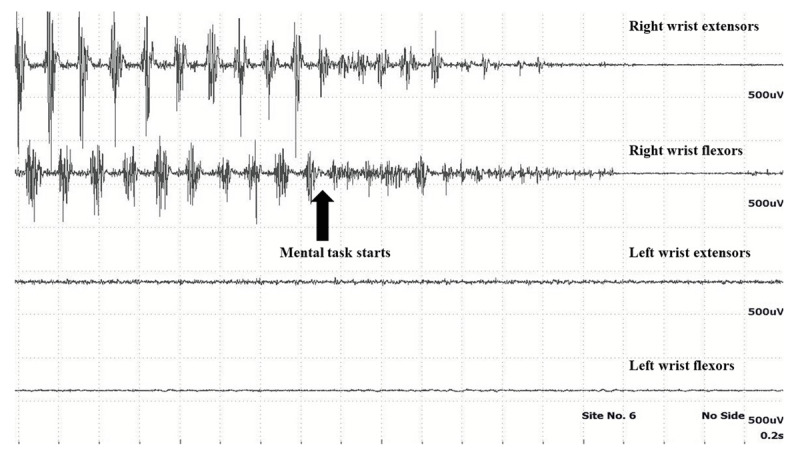
Multichannel surface EMG showing distractibility while performing mental task in a patient with functional tremor (from authors’ archive).

During the initial baseline recording, the EMG bursts are observed for any variability in the pattern of muscle activation, the frequency, burst duration and the amplitude which suggests a possibility of functional tremors. The patient is asked to tap the contralateral unaffected limb (finger or foot) at a different frequency. The original tremor frequency may change to the tapping frequency which is called as “entrainment” (Figure 2, Video 3). More often the tremor frequency, amplitude and pattern changes suggesting distractibility (Figure 3) [18]. Poor tapping may itself point towards a functional cause. Mental subtraction, ballistic movements, precise motor tasks may also interrupt the tremors by causing distraction [10]. Ballistic movements of the unaffected contralateral limb transiently stop the tremor or causes more than 50% reduction in the tremor amplitude in majority of the trials [18, 49]. It should be emphasized that during the entrainment testing using sEMG, a significant coherence observed at the tapping frequency between the limb with functional tremors and the tapping limb is highly suggestive of functional tremors. Such high coherence is not observed in organic tremors [50].

**Video 3:** Demonstration of entrainment (voluntary movements of left hand cause the frequency and pattern of movement to change in the right hand).

Loading the tremulous limb with 500 g weight causes the tremor amplitude to increase in patients with functional tremor. Whereas, in organic tremor, the tremor amplitude may reduce or remain unchanged. This change in the amplitude of the tremor after loading has a very high specificity (92%) but has very low sensitivity (22%) [51]. The tremor frequency usually remains unchanged both in patients with organic and functional tremor, however, if the tremor frequency increases then it is highly suggestive of functional tremors [48]. The tremor amplitude may increase by more than 130% on 500 g weight loading which is highly suggestive of functional tremor [18]. However, occasionally this increase in tremor amplitude on weight loading can also be observed in patients with essential tremor and Parkinson’s disease tremor [45].

In a pilot study, a battery of electrophysiological tests was used to differentiate between organic and functional tremors [18]. These tests included changes in tremor amplitude on weight loading, changes in tremor frequency with tapping, pause of tremor during ballistic movements, coherence analysis and co-activation sign. A significant difference was noted on group comparisons with excellent sensitivity and specificity, however, the sensitivity and specificity of all separate tests varied widely between 33% to 77% and 84% to 100%. Hence, they concluded that a combination of electrophysiological tests is essential to distinguish between functional and organic tremor with excellent sensitivity and specificity [18].

Bereitschaftspotential (BP) has been used to demonstrate the functional nature of the palatal tremor in few cases [52]. BP in these cases were recorded approximately 800 msec prior to the onset of the palatal movement. The surface electrodes were placed on the soft palate along with simultaneous EEG recording [52].

### Functional myoclonus

**Figure 4:**
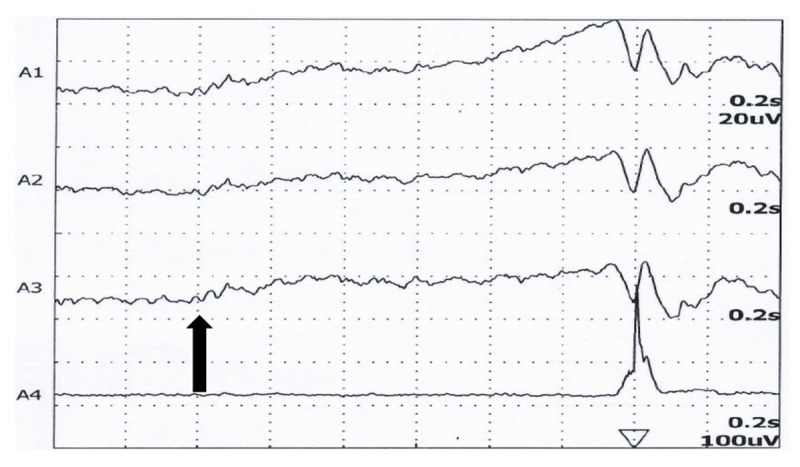
Bereitschaftspotential in a patient with functional myoclonus. Arrow indicates the onset of BP about 1200 msec before the onset of movement (from authors’ archive).

The clinical signs that help in suspecting functional myoclonus includes distractibility, variability and its progression. Multichannel sEMG, conventional electroencephalography (EEG), Jerk-Locked-Back-Averaging (JLBA), somatosensory evoked potentials (SEP), and C-reflex studies are used to characterize the myoclonus. In comparison to cortical myoclonus, functional myoclonus does not have giant SEP, enhanced LLR or C-reflex nor any EEG correlate of the myoclonus. Simultaneous recording of the EEG and EMG helps in determining whether the myoclonus is preceded by EEG activity that strongly suggests cortical origin of the myoclonus. BP can be demonstrated in functional myoclonus (Figure 4). Functional myoclonus that occurs more frequently with a duration less than 2 seconds between the myoclonic jerk makes it difficult to elicit BP. In addition, BP can be demonstrated in spontaneous myoclonus but not in action induced myoclonus [10]. In a study involving 29 patients with functional myoclonus, 5 with organic myoclonus, 14 with Tourette syndrome, and 25 healthy subjects, BP was observed significantly in patients with functional myoclonus both during the spontaneous jerks (86%) and during intentional wrist extension (41%). However, in patients with organic myoclonus, BP was not observed during spontaneous jerks but was observed in intentional wrist extension (100%) [27]. Absence of BP prior to the volitional/intentional movement has a sensitivity of 0.59, specificity of 0.98 and a positive likelihood ratio of 25 in diagnosing functional myoclonus [27]. In addition, BP occurs significantly much earlier in patients with functional myoclonus compared to Tourette syndrome.

A triphasic pattern of agonist and antagonist muscle activation is seen in functional myoclonus [47]. The EMG burst duration is also variable with prolonged EMG burst duration (>200 msec). Functional myoclonus can be stimulus sensitive also. Various stimuli have been used to elicit functional myoclonus such as tapping with tendon hammer, electrical stimulation of the digit or by using loud noise [49]. The onset latency in response to stimulation is more than 100 msec in functional myoclonus [41]. In addition, they have variable latencies, variable patterns of muscle recruitment and movements tend to reduce significantly with repeated stimulation [53]. Presence of any or all the following electrophysiological features: giant SEP, C-reflex and an abnormal EEG rules out functional myoclonus.

Spinal segmental myoclonus is a type of myoclonus that involves one or a few contiguous spinal myotomes causing axial jerks of the trunk without involvement of the cranial nerve innervated muscles. Propriospinal myoclonus is a rare type of spinal myoclonus, that propagates slowly both up and down the spinal cord causing repetitive arrhythmic flexion and/or extension of the trunk and may sometime involve the neck, knees, hips etc [54]. Three types of propriospinal myoclonus have been identified that includes idiopathic, symptomatic and functional. Functional propriospinal myoclonus accounts for more than half the cases reported [55]. Electrophysiologically, the propriospinal myoclonus is characterized by long duration EMG bursts (~150 to 450 milliseconds or even longer), efferent volley conduction velocity of 5 to 15 m/sec, cranio-caudal propagation of myoclonus that is limited to the spinal cord, inconsistent pattern of muscle activation and presence of BP [56, 57].

### Functional dystonia

Although a number of electrophysiological tests are available to distinguish organic tremors/myoclonus from functional tremors/myoclonus, there are no such tests that can sufficiently distinguish organic dystonia from functional dystonia [2]. It is a much less understood condition and is hence difficult to differentiate it from organic dystonia. Also, it is difficult to perform and interpret electrophysiological tests in these patients. Fahn and Williams initially proposed the diagnostic criteria of functional dystonia as early as 1988 [39].

EMG studies have been used in research but are not useful clinically [58]. Co-contraction testing may be helpful in differentiating functional from organic dystonia but not at an individual patient level [59]. Continuous co-contraction is usually a feature of functional dystonia, whereas phasic co-contraction with variable duration is seen in organic dystonia. Presence of geste anatagoniste, null point will help in differentiating dystonic tremor from functional dystonia.

The R2 blink reflex recovery cycle is an electrophysiological measure of the brainstem interneuron excitability. This has been found to be abnormally enhanced in organic blepharospasm suggesting reduced brainstem inhibition, whereas it is normal in presumed functional blepharospasm [60]. An abnormal R2 index can identify clinically organic blepharospasm with a sensitivity of 100% and specificity of 90% [60].

The plasticity measured by paired associative stimulation (PAS) was found to be abnormally high in organic dystonia while it was normal in functional dystonia. In addition, enhanced facilitation of the motor evoked potentials (MEP) was found in patients with organic dystonia but not in functional dystonia [34]. Similar results were also observed in other studies also [61]. Functional dystonia and organic dystonia’s share similar electrophysiological features with respect to TMS. Patients with organic and functional dystonia have significantly reduced SICI, LICI and SP compared to healthy controls [33, 34, 61]. In addition, the short-latency afferent inhibition (SAI) and long-latency afferent inhibition (LAI) are normal in patients with functional dystonia. However, the plasticity as measured by PAS is abnormally high in patients with organic dystonia compared to functional dystonia [34].

Electrophysiologically, reaction time and co-contraction has been shown to differentiate organic from functional dystonia during voluntary movements at a group level, but not suitable at individual level [59]. Currently there are no robust electrophysiological tests available for differentiating functional from organic dystonia. EMG channels should include the agonist-antagonist muscles to look for co-contraction and the unaffected muscles to look for overflow muscle activation. Though co-contraction is a feature of organic dystonia, it may also be a feature of functional dystonia.

### Functional Tics

Gilles de la Tourette syndrome (GTS), is a neuropsychiatric disorder characterized by multiple motor, vocal/phonic tics and/or obsessive-compulsive or attention deficitand hyperactivity disorders [5]. These phenomenologies either occur singly or in variable combinations. BP is not uncommon in patients with functional tics [62]. It is usually absent in tic disorders, if present a late BP is observed (1500 – 500 msec prior to the movement). BP can sometimes help in differentiating organic tics from functional jerks. The duration of BP in tic disorders is shorter in comparison to functional jerks [27]. BP in functional tics have a significantly earlier onset compared to Gilles de la Tourette syndrome. In addition, BP prior to voluntary or intentional movements is absent in patients with functional tics [27]. Demonstration of consistency in the pattern of muscle activation on sEMG recording favours organic tic disorder rather than functional [62]. The details of the various electrophysiological methods and their findings in various FMDs is summarized in Table 1. In addition, Table 2 lists the electrophysiological methods used in patients with tremors and myoclonus.

**Table 1:** Electrophysiological characteristics of functional tremors, functional myoclonus, functional dystonia and functional tics.


TYPE OF FMD	ELECTROPHYSIOLOGICAL METHODS	ELECTROPHYSIOLOGICAL FINDINGS

Functional Tremor	Multichannel surface electromyography (sEMG)	Prolonged EMG burst duration Varying EMG burst duration Increase in tremor EMG amplitude by more than 130% on peripheral weight loading Variability in tremor frequency, pattern and amplitude on distraction Can be entrained Co-activation sign Pause or reduced tremor amplitude on ballistic movements

	Coherence analysis	Significant coherence between the “involuntary” and “voluntary” movements

	Accelerometry	Similar to sEMG

Functional Myoclonus	Multichannel surface electromyography (sEMG)	Triphasic pattern on agonist and antagonist muscle activation Variable latencies to stimulation (more than 100 msec) Prolonged EMG burst duration in propriospinal myoclonus (150–450 msec or longer)

	Bereitschaftspotential (BP)	Present. Absence of BP during the intended movement

	Evoked potentials	No Giant SEP

	LLR or C-reflex	Absent

	Jerk locked back averaging	No cortical potential

	EEG	Normal

Functional Dystonia	Multichannel surface electromyography (sEMG)	Co-contraction of the muscles

	R2 Blink Reflex Recovery curve and R2 index	Normal

	Transcranial magnetic stimulation	Reduced SICI, LICI and SP Normal SAI and LAI Normal plasticity as measured by PAS

Functional Tics	Multichannel surface electromyography (sEMG)	Inconsistent pattern on muscle activation

	Bereitschaftspotential (BP)	Not uncommon Early BP Absent BP prior to voluntary or intentional movements


BP-Bereitschaftspotential, EEG-Electroencephalography, EMG-Electromyography, LICI-Long interval intracortical inhibition, LAI-Long latency afferent inhibition, LLR-Long loop reflex, SAI-Short latency afferent inhibition, SEP-Somatosensory evoked potential, SICI-Short interval intracortical inhibition, SP-Silent period.

**Table 2:** Electrophysiological methods for the evaluation of tremor and myoclonus.


ELECTROPHYSIOLOGICAL METHODS	TREMOR	MYOCLONUS

Multichannel sEMG	Yes	Yes

Coherence analysis	Yes	Yes

Electroencephalography (EEG)	No	Yes

Jerk locked back averaging (EEG-EMG back averaging)	No	Yes

Somatosensory evoked potentials (SSEP)	No	Yes

Bereitschaftspotential	No	Yes


### Limitations of electrophysiological testing in FMD

Though electrophysiology aids in diagnosis of FMDs, there are certain limitations. Entrainment sometimes may not be observed in functional tremors [63]. These patients can maintain two tremors in different limbs with different frequencies. The EMG burst duration is usually prolonged in functional tremors compared to organic tremors. However, the burst duration is variable according to the frequency of the tremor. The higher the frequency, the shorter the burst duration. In addition, in patients with long standing functional tremors and in those with bilateral functional tremors, distractibility may not be observed. Electrophysiology can sometimes be challenging in differentiating organic and functional propriospinal myoclonus [64]. Current evidence however suggest that propriospinal myoclonus is functional in origin.

## Conclusions

Functional movement disorders are one among the debilitating neurological disorders that greatly limit the psychological, social, emotional well-being and reduces the quality of life of these patients. Diagnosis is difficult as there are no characteristic serological and imaging biomarkers. Hence the diagnosis is mainly clinical and supplemented by detailed electrophysiological tests. Among the various electrophysiological tests available, multichannel sEMG along with accelerometry, coherence analysis, EEG, SEP, C-reflex, bereitschaftspotential, and TMS are useful in differentiating FMD from organic movement disorders. The choice of electrophysiological test to be done depends on the phenomenology of FMD. These electrophysiological studies improve the diagnostic accuracy of FMD and should be considered as an extension of the clinical examination.
